# Individual Attachment Style Modulates Human Amygdala and Striatum Activation during Social Appraisal

**DOI:** 10.1371/journal.pone.0002868

**Published:** 2008-08-06

**Authors:** Pascal Vrtička, Frédéric Andersson, Didier Grandjean, David Sander, Patrik Vuilleumier

**Affiliations:** 1 Swiss National Center for Affective Sciences, University of Geneva, Geneva, Switzerland; 2 Laboratory for Neurology & Imaging of Cognition, Department of Neurosciences, Clinic of Neurology, University Medical Center of Geneva, Geneva, Switzerland; 3 Department of Psychology, FPSE, University of Geneva, Geneva, Switzerland; Claremont Graduate University, United States of America

## Abstract

Adult attachment style refers to individual personality traits that strongly influence emotional bonds and reactions to social partners. Behavioral research has shown that adult attachment style reflects profound differences in sensitivity to social signals of support or conflict, but the neural substrates underlying such differences remain unsettled. Using functional magnetic resonance imaging (fMRI), we examined how the three classic prototypes of attachment style (secure, avoidant, anxious) modulate brain responses to facial expressions conveying either positive or negative feedback about task performance (either supportive or hostile) in a social game context. Activation of striatum and ventral tegmental area was enhanced to positive feedback signaled by a smiling face, but this was reduced in participants with avoidant attachment, indicating relative impassiveness to social reward. Conversely, a left amygdala response was evoked by angry faces associated with negative feedback, and correlated positively with anxious attachment, suggesting an increased sensitivity to social punishment. Secure attachment showed mirror effects in striatum and amygdala, but no other specific correlate. These results reveal a critical role for brain systems implicated in reward and threat processing in the biological underpinnings of adult attachment style, and provide new support to psychological models that have postulated two separate affective dimensions to explain these individual differences, centered on the ventral striatum and amygdala circuits, respectively. These findings also demonstrate that brain responses to face expressions are not driven by facial features alone but determined by the personal significance of expressions in current social context. By linking fundamental psychosocial dimensions of adult attachment with brain function, our results do not only corroborate their biological bases but also help understand their impact on behavior.

## Introduction

Since its description four decades ago, attachment theory [1], [2] has become one of the most important frameworks for understanding affect regulation in social interactions [3], [4]. Initially grounded on child-mother relationships [1], [2], the functions of attachment were first related to the regulation of proximity-seeking behavior, with the goal to obtain protection and care from another person (an attachment figure). Because of a consistent pattern of engagement of attachment processes during a range of situations from infancy to adulthood, behavioral researchers have suggested that attachment models may become part of general interpersonal schemata for an individual, which will support social development and influence thoughts, feelings, and behavior throughout the lifespan [3]. Thus, attachment theory has been extended to adults to describe affective responses in the context of various relationships, particularly with romantic partners or close friends [5]. Moreover, subsequent work demonstrated that attachment style in adults may also predict behaviors and judgments regarding unfamiliar persons [6], [7], as well as social and emotional processes in various tasks [3]. These findings are consistent with the idea that people use general schemata of interpersonal relationships at different levels of representations from parents and close partners through to less familiar persons [8]. Therefore, although the exact links with developmental aspects of the attachment system are still unclear [9], it is generally thought that attachment style in adults entails fundamental individual biases that can influence how people perceive and respond to social information in a wide range of relationship contexts.

Following earlier studies on children and parents, classic models have distinguished between three main prototypes of attachment style in adults: secure, anxious, and avoidant [5]; whereas subsequent work suggested the existence of four [10] or even five [11], [12] distinct styles. More recent models proposed instead that these different styles might be mapped on two basic dimensions defined by orthogonal axes of anxiety and avoidance, with the secure style corresponding to both low anxiety and low avoidance [10]. Individuals with an anxious attachment style (AXS) tend to perceive others as unresponsive or inconsistent, worry about being rejected, and show heightened vigilance to signs of support or hostility; whereas individuals with an avoidant attachment style (AVS) prefer being distant and detached from others, report no need for close relationships, and tend to distrust affective signals from others. By contrast, individuals with a secure attachment style (SAS) report positive and trustful interactions with others. More generally, it has been shown that attachment style may shape the way in which individuals appraise social information in a variety of conditions [6], [7], [13], including during interactions with strangers [8], [14]. For instance, attachment styles can determine whether feedback messages given by partners are perceived as supportive or not [15], predict the experience of conflict in interpersonal contexts [16], [17], and influence the perception of emotional expressions in pictures of unknown faces [6], [7]. Adult attachment style is also related to individual differences in cooperativeness, reward dependence, and novelty seeking [11], [18]. Based on behavioral studies, researchers have proposed that AXS might reflect higher states of vigilance and sensitivity to socially significant cues, while AVS might involve either deactivation or inhibition of affective responses to interpersonal situations [3], [6], [17]. However, although attachment theory has generated a rich body of research in social and clinical psychology, the neural bases of these individual differences remain unknown.

Here we developed a new fMRI paradigm to determine the influence of the three classic types of adult attachment style (secure, anxious, or avoidant) on appraisal of social cues in the human brain. We tested whether individual differences in attachment style are linked to relative decreases or increases in the activity of brain regions associated with social and emotional processing, and whether such effects might depend on the personal significance of social signals, by presenting faces with expressions that could be perceived as either friendly (supportive) vs unfriendly (unsupportive or hostile). Previous neuroimaging studies concerning attachment have focused on particular relationships such as maternal and romantic love, without considering individual attachment style [19], [20], and reported that pictures of loved individuals deactivate the amygdala and activate the striatum, two brain regions critical for affective processing and reinforcement [21], [22]. Other studies found activation of amygdala [23] and medial prefrontal cortex [24] to sentences or scenarios with attachment-related meaning, but did not examine the differential effects of classic attachment styles on the perception of social cues with different affective meanings. Since adult attachment can shape emotional responses to socially relevant signals, our study specifically aimed at comparing the influence of distinct attachment styles on the processing of negative and positive social stimuli in attachment-related contexts. We hypothesized that individual differences in attachment styles should modulate activation patterns in brain circuits known to mediate social perception and behavior, particularly in emotional limbic regions such as the amygdala, ventral striatum, and ventromedial prefrontal cortex. Alternatively, since attachment style can also influence the formation of “mental models” of others [10], [25], [26], it might primarily modulate the recruitment of higher-level cortical regions associated with mentalizing and theory of mind such as superior temporal sulcus (STS) and anterior cingulate cortex [27].

To generate context-specific appraisal of social signals in our study, participants saw faces with smiling or angry expressions in a pseudo-game context, while they underwent event-related fMRI scanning (see Methods). Because attachment style is critically related to the way people evaluate signs of alliance and opposition during social interactions [17], we systematically manipulated the social significance of these facial expressions to elicit a perception of either supportive or unsupportive partners. We took advantage of the fact that smiling or angry expressions can have very different meanings based on current context. For instance, a smile may be perceived as praising an accomplishment or mocking a failure. Likewise, angry expressions may signal reproach or frustration. By inducing specific social meanings for these facial expressions presented in different scenarios, we could test the hypothesis that attachment style might influence affective appraisal of social facial signals, as suggested by previous behavioral studies [6], [7], [15].

In accord with these predictions, our results show for the first time that adult attachment style modulates neural responses to the perceived social meaning of facial expressions in brain regions critically associated with affective processing and learning, namely ventral striatum and amygdala. Furthermore, we show that the two dimensions of avoidance and anxiety produce distinct effects in these two regions, and thus appear sufficient to account for the effects of secure attachment, in agreement with previous theoretical proposals [10].

## Results

**Figure 1:**
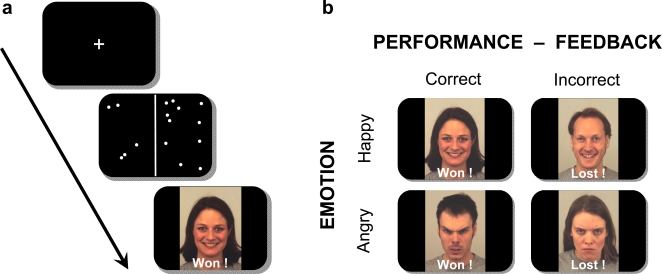
Illustration of the paradigm and the four different feedback conditions. a) Illustration of the paradigm: Participants first saw a central fixation cross, followed by the dot-counting task (0.5 sec), where they had to indicate which side of the screen contained more dots (right vs left). Following each response, a visual feedback was shown (1.5 sec), composed of a word together with a face. b) Illustration of the four different feedback conditions: two socially “congruent” (Smiling Face on WON trial, Angry Face on LOST trials) and two socially “incongruent” (Smiling Face on LOST trial, Angry Face on WON trial) combinations were possible. Four different face identities (2 female and 2 male) were used in each of these 4 conditions. See Materials and Methods for further details.

Participants were presented with smiling or angry faces accompanying a feedback message about their current performance in a difficult perceptual task (Fig. 1a). On each trial, they first saw a visual array in which they had to judge the number of dots. Feedback was then displayed, consisting of a word indicating actual performance (“WON” or “LOST”) together with a face (Fig. 1a). Critically, the face could have either a smiling or angry expression, and could appear on either a WON or LOST trial (half each). This resulted in four feedback types, with two “congruent” and two “incongruent” conditions (Fig. 1b): Smiling Face on WON trial (SF-W) or LOST trial (SF-L), Angry Face on LOST trial (AF-L) or WON trial (AF-W). Participants were told that these faces were from two different groups receiving points based on their performance, such that they could be perceived as either allied partners (SF-W and AF-L; congruent conditions) or opponents (SF-L and AF-W; incongruent conditions) in a virtual game context (see Methods). After fMRI scanning, a series of questionnaires was given to assess attachment style [28] as well as other affective traits and debriefing measures (see Methods). We used a standardized scale, the Adult Attachment Questionnaire (AAQ; [28]), which provides three scores for each individual, corresponding to the relative strength of each of the three classic attachment styles: two indices for avoidant (AVS) and anxious (AXS) attachment, and one global score for secure attachment (see Methods for more details). Combinations of the first two scores also provide a reliable measure along two separate dimensions of avoidance and anxiety [29]. Individual differences in attachment style did not influence performance on the dot counting task (accuracy and reaction times). Debriefing questionnaires after scanning indicated that participants were motivated by the task and reported genuine affective reactions to facial expressions seen in different feedback context (see Methods).

### Main effects of feedback and expression

First, we examined brain responses to each feedback type, regardless of the concomitant face expression. Success feedback (all WON>LOST trials) activated a widespread network in bilateral basal ganglia, left orbitofrontal cortex (OFC), anterior cingulate, and dorsolateral prefrontal areas (see Table 1), consistent with a general role of these regions in monitoring outcomes and rewards [22], [30]. Conversely, error feedback (LOST>WON) activated retrosplenial cortex and right insula (see Table 1), consistent with previous studies on evaluation of negative events [22]. We also examined brain responses to each emotional face expression (smiling>angry or vice versa), regardless of concomitant success feedback. However, the latter contrasts did not reveal any region showing only main effects of expression at standard statistical thresholds (p<.001).

**Table 1:** Brain areas activated in the main contrasts between conditions, listed with peak coordinates and best estimates of anatomical location.

*Brain Area*	Main Contrasts						
	*BA*	*Voxel*	*X*	*Y*	*Z*	*Z-Value*	*P-Value*
**Won>Lost**							
OFC left	11	36	−36	39	−15	4.87	P<0.001
Caudate right		5	18	21	18	3.8	P<0.001
Nucleus accumbens right		6	12	12	−6	3.55	P<0.001
Putamen left		5	−24	9	0	3.48	P<0.001
dlPFC right	46	100	30	18	42	4.77	P<0.001
dlPFC right	46	41	33	51	24	3.92	P<0.001
dlPFC left	45/46	12	−42	48	24	4.07	P<0.001
dlPFC left	9	56	−30	15	48	3.99	P<0.001
Dorsal ACC right	25	40	15	−6	48	3.92	P<0.001
Hippocampus right		34	27	−21	−24	4.1	P<0.001
Occipital cortex left	17	6	−15	−108	9	3.7	P<0.001
Angular gyrus right	39	13	45	−63	33	3.66	P<0.001
**Lost>Won**							
Retrosplenial cortex left	26	18	−3	−36	15	3.3	P<0.001
Supramarginal gyrus left	40	5	−63	−48	30	3.15	P<0.001
Insula right		5	42	−3	−3	2.81	P<0.002
**Smiling>Angry Faces with Won Feedback**							
**(SF-W>AF-W)**							
OFC left	11	7	−24	48	−3	4.36	P<0.001
Parietal cortex right	7	25	15	−63	63	3.79	P<0.001
ACC right	24	7	9	33	18	3.72	P<0.001
Supramarginal gyrus right	40	9	51	−42	57	3.66	P<0.001
Ventral striatum left		5	−24	18	−12	3.46	P<0.001
Supramarginal gyrus left	40	5	−45	−48	57	3.41	P<0.001
Occipital cortex right	18	9	3	−90	12	3.36	P<0.001
**Angry>Smiling Faces with Lost Feedback**							
**(AF-L>SF-L)**							
Amygdala left (dorso-medial)		27	−12	−3	−15	4.62	P<0.001
Post hippocampus left		14	−33	−33	−6	3.37	P<0.001
Insula right		6	39	0	−18	3.04	P<0.001
**Angry>Smiling Faces with Won Feedback**							
**(AF-W>SF-W)**							
Supramarginal gyrus left	40	14	−48	−42	36	3.92	P<0.001
STS right	21	12	48	−39	0	3.86	P<0.001
Inferior frontal gyrus left	44	22	−45	9	24	3.63	P<0.001
**Smiling>Angry Faces with Lost Feedback**							
**(SF-L>AF-L)**							
STS left	21	15	−48	−39	−6	4.2	P<0.001
Angular gyrus right	39	7	60	−54	36	3.71	P<0.001
Inferior frontal gyrus left	44	16	−54	21	33	3.61	P<0.001
Parietal cortex right	40	23	54	−57	48	3.52	P<0.001
Occipital cortex left	17	39	−15	−105	6	3.48	P<0.001

Coordinates are given in MNI space. Activation sites were determined on the basis of the average anatomical MRI images of our 16 subjects. BA = Brodmann's area, OFC = Orbitofrontal cortex, dlPFC = Dorsolateral prefrontal cortex, ACC = Anterior cingulate cortex, STS = Superior temporal sulcus.

### Social meaning of congruent positive feedback: Responses to perceived support

**Figure 2:**
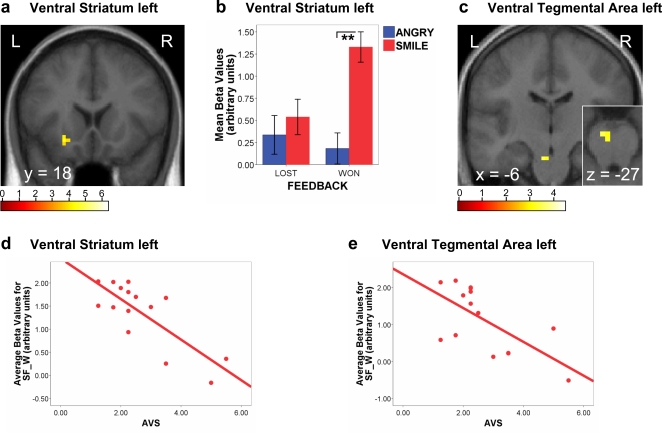
fMRI and attachment style results for the social support (SF-W) condition. a) Statistical parametric map for Smiling vs Angry expressions in success feedback context (contrast SF-W>AF-W), showing selective activation in left ventral striatum (xyz = −24 18 −12, z-score = 3.46, p<.001). b) Activation of the left ventral striatum cluster is plotted across all conditions (mean beta values±inter-subject s.e.m.), showing significant increases to Smiling Faces only when paired with success feedback (t = 5.21, p<.001), not when paired with error feedback (SF-L>AF-L, p = .54). c) Statistical parametric map for the whole-brain regression analysis between AVS and activation to Smiling Faces in the success feedback condition (contrast SF-W>others), showing a selective effect in the left ventral tegmental area (VTA; xyz = −6 −18 −27; z-score = 2.80). The small inset panel shows a horizontal section through the midbrain at the level of VTA. d) Negative correlation between avoidant attachment scores (AVS) and activity in the ventral striatum cluster (beta values relative to baseline) for Smiling Faces in the success feedback condition (SF-W; Pearson r = −.787, p<.001). e) Activity in the VTA cluster for condition SF-W was also inversely correlated with AVS (Pearson r = −.706, p = .003). L = Left, R = Right. ** = p<.001.

Next, we examined the different activation patterns evoked by the same expression in different feedback contexts, focusing first on the congruent conditions. When comparing responses to smiling vs angry faces on WON trials (SF-W>AF-W), a condition corresponding to the perception of social support, we found selective increases in left ventral striatum (Fig. 2a) and left OFC (Table 1). These activations were not due to success alone, because performance feedback was positive (WON) in both conditions. No such increases were found for smiling faces paired with the negative (LOST) feedback (Fig. 2b). Hence, these responses reflected the social value of reward, rather than reward or facial expression only. A repeated-measure ANOVA on the average parameter estimates of activity (betas) extracted from left ventral striatum confirmed a significant interaction between face expression and performance success (F_1,15_ = 5.99, p = .027).

We then examined the relation between left ventral striatum activity and attachment indices, using parameter estimates of activity for this cluster in each trial type. This showed a strong negative correlation between the magnitude of response to smiling faces with success feedback (SF-W) and the degree of avoidant attachment (AVS, r = −.787, p = .001; Fig. 2d), but no such relation for other conditions, including success feedback with angry faces (AF-W). No significant relation was found for OFC. Other attachment indices did not correlate with neural activity in any of these areas.

These results were confirmed by a whole-brain multiple regression analysis of the response elicited by smiling faces with success feedback (SF-W), using the three attachment scores as separate parametric factors in a single SPM design, in order to test for any voxels throughout the brain where activation in this social reward condition (SF-W) varied as a function of each attachment style. To rule out that any correlation with SF-W would be partly confounded by an inverse correlation with AF-W, our SPM regression analysis was performed on the contrast of SF-W versus the other three conditions (SF-W>others), rather than on the contrast of SF-W>AF-W as used above (but results of these two analyses were in fact similar). In addition to the left ventral striatum, this whole-brain regression analysis revealed a highly significant negative relation (p<.001) between AVS and activity in anterior insula and left midbrain, overlapping with the ventral tegmental area (VTA, Figs. 2ce and Table 2). Thus, higher AVS scores predicted lower activation in several brain regions associated with dopaminergic function and reward, including both ventral striatum and VTA [22], [30].

**Table 2:** Brain areas activated in parametric correlation analyses using attachment scores from the Adult Attachment Questionnaire (secure = SAS, anxious = AXS, avoidant = AVS).

*Brain Area*	Correlations						
	*BA*	*Voxel*	*X*	*Y*	*Z*	*Z-Value*	*P-Value*
**SF-W>others×AVS (negative)**							
Anterior insula left		12	−27	21	−15	3.23	P<0.001
Ventral striatum left		10	−24	18	−12	3.04	P<0.001
Ventral tegmental area left		3	−6	−18	−27	2.8	P<0.003
**SF-W>others×SAS (positive)**							
Anterior insula left		19	−27	21	−15	3.2	P<0.001
Ventral striatum left		12	−24	18	−12	3	P<0.001
**AF-L>others×SAS (negative)**							
Amygdala left		9	−24	−9	−21	3.35	P<0.001
Medial thalamus left		4	−9	−15	−30	3.21	P<0.001
**AF-L>others×AXS (positive)**							
Amygdala left		10	−24	−9	−21	3.3	P<0.001
Medial thalamus left		4	−9	−15	−30	2.91	P<0.002
**AF-W>others×AVS (positive)**							
Retrosplenial cortex left	30	82	−12	−51	15	4.21	P<0.001
Insula right		16	42	18	0	3.98	P<0.001
Dorsal ACC left	11	6	−9	42	36	3.16	P<0.001
Ventral ACC left	32	9	−12	30	−9	3.07	P<0.001

Peak coordinates are given in MNI space and listed with best estimates of anatomical location. BA = Brodmann's area, STS = Superior temporal sulcus, VTA = Ventral tegmental area.

Conversely, the same regression analysis also revealed that high scores on the SAS dimension correlated positively with ventral striatum and insula activity (see Table 2). However, there was no significant correlation between SAS and VTA. Finally, no correlation was found for AXS scores and other personality factors related to anxiety (BIS/BAS, STAI-T) or more general affective traits (PANAS). This complementary correlation profile between AVS and SAS suggests that activation of reward-related regions to situations representing social support is associated with secure attachment style, whereas a lack of activation is associated with avoidant attachment.

### Social meaning of congruent negative feedback: Responses to perceived reproach

**Figure 3:**
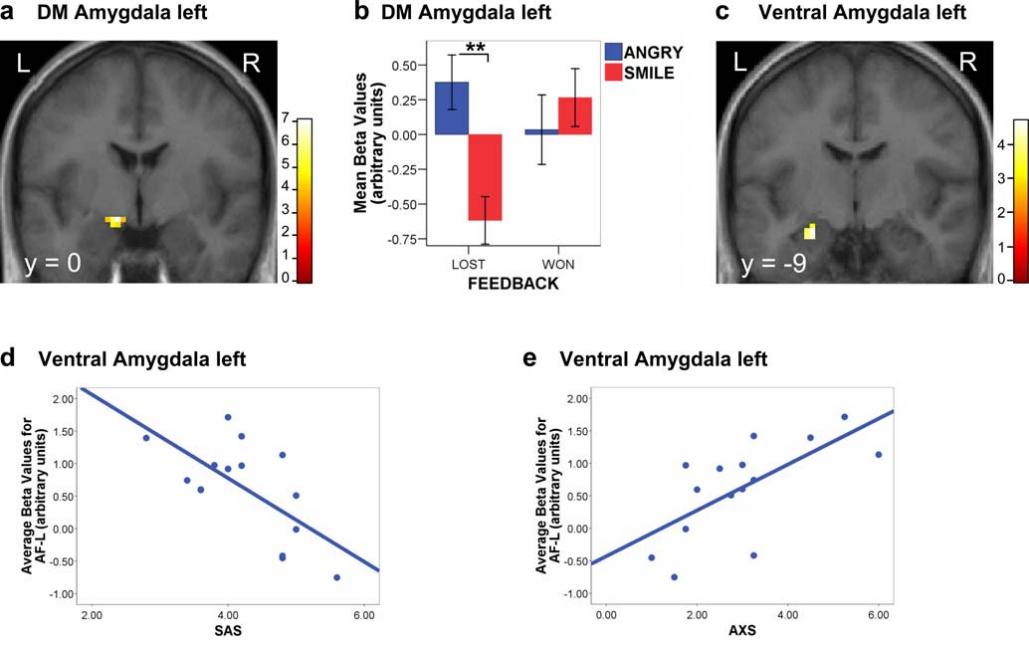
fMRI and attachment style results for the social punishment (AF-L) condition. a) Statistical parametric map for Angry vs Smiling expressions in error feedback context (contrast AF-L>SF-L), showing activation in left dorso-medial amygdala (xyz = −12 −3 −15, z-score = 4.62, p<.001). b) Activation for this amygdala cluster is plotted across all feedback conditions (mean beta values±inter-subject s.e.m), showing significant difference between Angry and Smiling faces with error feedback (t = 4.94, p<.001) but no significant difference with success feedback (p = .50). c) Statistical parametric map for the whole-brain multiple regression analysis between AXS and activation to Angry Faces in the lost feedback condition (contrast AF-L>others), showing a selective effect in left amygdala (xyz = −24 −9 −21; z-score = 3.30). d) Activity in this amygdala cluster (beta values relative to baseline) for Angry Faces in the lost feedback condition (AF-L) was negatively correlated with SAS (Pearson r = −.655, p = .008). e) Activity in the same amygdala cluster for condition AF-L was also positively correlated with AXS (Pearson r = .668, p = .006). L = Left, R = Right. ** = p<.001.

Brain responses to angry expressions also differed as a function of feedback and attachment style. When comparing angry vs smiling faces in LOST trials (AF-L>SF-L), a condition meant to evoke signs of reproach or social punishment, we found significant activation in left dorsal amygdala (Fig. 3a), as well as left hippocampus and right insula (see Table 1). Amygdala activation was not due to incorrect performance alone, because error feedback (LOST) was similar in both conditions. In addition, no such increase was found for angry vs smiling faces in WON trials (Fig. 3b). Again, these responses reflected the social meaning of feedback, rather than loss or facial expression per se. Accordingly, a repeated-measure ANOVA on the average parameter estimates of activity (betas) from this amygdala cluster confirmed a significant interaction between face expression and feedback type (F_1,15_ = 8.19, p = .012). When testing for an association with attachment indices, we found a specific negative relation between the dorsal amygdala response to angry faces with error feedback (AF-L) and the degree of secure attachment (SAS, r = −.487, p = .033), but no such relation for the three other feedback conditions.

Again, we confirmed this correlation by a whole-brain multiple regression analysis of activation to angry faces with error feedback (AF-L>others), using attachment scores as separate parametric factors in a single SPM design (similar results were found using the contrast AF-L>SF-L). In keeping with the above, we found highly significant effects in the left amygdala (Fig. 3c). This region showed not only a strong positive correlation with AXS, but also a negative correlation with SAS, selectively for the AF-L condition in both cases (p<.001, Figs. 3de and Table 2). Similar correlations with AXS and SAS were also found in the left medial thalamus. No relation was found for the AVS dimension in this condition. Again, these correlations were specific to these two attachment indices, but not found for more general anxiety or affective measures (STAI-T, BIS/BAS, PANAS). This complementary correlation profile between SAS and AXS suggests that secure attachment is related to low anxiety as well as low avoidance, rather than to a single distinctive pattern of brain responses.

### Incongruent feedback trials: Responses to perceived social conflict

For completeness, we also examined brain responses to the two socially “incongruent” feedback conditions, corresponding to the perception of opponent faces (smiling on LOST trials, SF-L>AF-L; or angry on WON trials, AF-W>SF-W). These conditions elicited selective activations in the left and right superior temporal sulcus (STS), respectively (see Table 1). However, these increases in STS did not correlate with attachment traits.

At a lower threshold, we also found a selective activation in the rostral ventral anterior cingulate cortex (vACC, BA 32, xyz = 9 54 3, z = 2.50, p <.006) for angry opponent faces (AF-W>SF-W), consistent with previous reports that ACC might be involved in conditions of social rejection and conflict [31]–[33]. Moreover, a whole-brain multiple regression analysis with attachment indices revealed a positive correlation between activation in these medial prefrontal regions to SF-W and AVS (vACC: xyz = −12 30 −9, z-score = 3.07, p<.001; r = 0.530; MPFC: xyz = −9 42 36, z = 3.16, p<.001; r = 0.757). No effect was found for the SF-L condition.

## Discussion

Our study provides several new results. Firstly, using a pseudo-social interaction paradigm, we show that brain responses to facial expressions are strongly modulated by the perceived social meaning induced by the current context. Smiling faces enhanced activation in the ventral striatum and related regions only when associated with positive feedback, whereas angry faces increased activation in amygdala only when associated with negative feedback. This indicates that responses in both striatum and amygdala were influenced by the social relevance of rewarding and punishment signals expressed by faces, respectively. While many studies have shown activation in striatum and OFC to various types of rewards such as gains or food, a few others have reported activation in the same regions to smiling or attractive faces [34], [35]. Here we found that such responses were not driven by facial features alone, but reflected the social meaning of a smiling expression, i.e., when perceived as rewarding current performance and as congruent with task-goals. Similarly, while several studies have reported activation of the amygdala to angry or negative facial expressions, here we show that this response may not be automatic and driven by specific facial features [36] but determined by the personal significance of perceived anger.

These findings provide support for the importance of appraisal of personal relevance in emotional processing [21], [37]. Moreover, these data also demonstrate that participants were highly motivated by the task (as also confirmed during debriefing). Indeed we observed a reliable main effect of positive (WON) or negative (LOST) feedback in regions associated with reward and motivation processes, including basal ganglia, OFC, and dorsolateral prefrontal cortex for WON trials, as well as retrosplenial cortex and right insula for LOST trials [22]. By contrast, we did not find main effects of facial expressions (smiling or angry), but only interactions of expressions with feedback context that confirm that our task induced specific social appraisals as a function of the pseudo-game context. In other words, the brain response to a visually similar facial expression was crucially dependent on current task-goals and feedback congruency, since the social significance of smiles on SF-W trials (praising success) clearly differed from SF-L trials (mocking a failure), whereas anger also differed between AF-L (reproach or punishment) and AF-W trials (conflict or frustration), resulting in distinct patterns of brain responses.

Secondly, and most importantly, we found that individual differences in adult attachment style strongly modulated responses to facial expressions in brain regions associated with affect and motivation; and that such modulation specifically concerned those conditions related to social appraisal. Our results therefore provide new support to the view that adult attachment style can shape how individuals perceive social information in various contexts, and converge with recent behavioral findings that it may modulate recognition judgments for emotional expressions in unfamiliar faces [6], [7]. These results also reveal that distinct neural substrates may underlie the two major dimensions of the attachment construct in healthy adults (anxiety and avoidance, as defined by self-report measures used here).

In the congruent feedback condition of social support (SF-W), we found that higher scores on attachment avoidance (AVS) predicted lower activation in brain regions linked to dopaminergic function and reward, including both ventral striatum and VTA [22], [30]. This correlation with AVS was highly specific for the SF-W condition representing a socially rewarding interaction (but not related to reward or positive affect of faces alone), supporting the hypothesis that individuals scoring higher on AVS may show reduced activation of affective processes in response to positive social signals [6], [38]. These findings are in line with behavioral evidence that people with high AVS tend to prefer physical and emotional distance from others, and usually do not seek social support [3], [39]. High AVS is also associated with greater self-reliance and a tendency to dismiss the benefits of group interactions [17]. Here we show that such tendencies to avoidant attachment may entail a relative down-regulation of reward-related activity in striatal circuits during socially reinforcing interactions, presumably underlying at least in part the relative impassiveness of individuals with high AVS to social rewards. Our results also provide a plausible substrate for behavioral observations that high AVS is negatively correlated with reward dependence [11], and add support to recent proposals that some forms of social avoidance may be associated with reduced positive experiences in social and non-social contexts [40].

On the other hand, we found that higher scores on anxious attachment (AXS) were correlated with selective increases in left amygdala responses to social signals of reproach or punishment (i.e. angry expressions combined with congruent negative feedback, AF-L). These data reveal that processing of socially aversive situations is specifically enhanced in brain systems associated with emotional arousal and fear [41] for people with higher anxious attachment. Because the amygdala is particularly implicated in processing self-relevant affective information [21], [42], our findings support the notion that a key aspect of anxious attachment may involve enhanced vigilance towards emotionally-significant social cues [3], [4], [17], [39]. These condition-specific responses also accord with the view that anxious attachment involves a “relation-specific anxiety” that is distinct from more general forms of anxiety or neuroticism [29]. In keeping with this, people with high AXS typically show increased monitoring and exaggerated appraisal of threats to the self, intensify negative emotional responses to emotional or social events, and unlike subjects with high AVS, tend to search more for external sources of support and comfort [3]. These results also converge with recent findings that greater amygdala responses to negative sentences may relate to attachment insecurity [23], although the latter study did not examine the distinct prototypes of attachment as here, but inferred more general attachment differences (secure or insecure) based on reaction times to the sentences (slow or fast).

Importantly, note that even though the AF-L condition represented negative social feedback, it was nevertheless congruent with the goals and expectations of participants on LOST trials, and thus did not correspond to a condition of social rejection or exclusion as implemented in other paradigms [31], [32]. Here, angry faces were perceived as in-group partners or allies who disapproved failures in the task and hence expressed punishment–a condition meant to activate the need for support in challenging or distressing situations that is intrinsic to anxious attachment style [3]. Accordingly, this condition evoked selective activation in the amygdala, rather than in anterior cingulate cortex as reported in previous studies where social rejection implied group exclusion or conflict [31]–[33].

In our study, the third prototype of adult attachment style (secure) did not exhibit any unique correlate for neural responses to the perceived social meaning of facial expressions, but mirrored the pattern found for AVS and AXS, respectively. Thus, high scores on SAS correlated positively with activation of the ventral striatum to rewarding smiles (SF-W) and negatively with activation of the amygdala to reproach faces (AF-L). These data therefore accord with the theoretical view that secure attachment may correspond to a combination of low anxiety and low avoidance, and add new neurobiological evidence in support of bi-dimensional models postulating that these two major components may account for the different categories of adult attachment style [10]. Critically, our fMRI results reveal that these two dimensions (anxious and avoidant attachment) have distinct neural bases in two key brain systems implicated in affect and motivation, centered on the amygdala and striatum, respectively. Both the striatum and amygdala play important roles for learning and predicting motivational outcomes in specific situational contexts, and might therefore be well suited for the establishment of idiosyncratic affective responses to social cues based on past experience or developmental history.

Thus, although the exact correspondence between developmental aspects of attachment initially described in infancy [1], [2] and attachment style in adults is still partly unclear [9], our results demonstrate that this social psychological construct taps specific affective processes, with distinct neural substrates, which can influence how people automatically perceive and respond to social signals in interaction contexts, beyond relationships with intimate partners or close personal acquaintances [3], [8]. In line with our findings, adult attachment has been shown to affect the recognition of emotional expressions in morphs of unfamiliar faces [6], [7], especially when such expressions are relevant to attachment concerns and interpersonal bonding [26], [43], as in our pseudo-social game paradigm. The current imaging findings that brain regions activated by face expressions are differentially modulated by individual attachment style provide new insights on the neurobiological underpinnings of these effects. More generally, unveiling such links between fundamental social dimensions and brain function may not only validate traditional psychosocial conceptualization but also help understand their impact on human behavior.

Activation of STS and MPFC were found only in incongruent feedback conditions corresponding to social opposition or confrontation (AF-W and SF-L), but did not correlate with attachment traits. STS is implicated in theory of mind and perception of intentionality [27], suggesting that participants were more inclined to imagine particular mental states or intentions for faces seen with incongruent feedback information. However, activity in STS did not appear to subtend differences in “mental models” of others that are typically associated with different attachment styles [5], [10], [25]. On the other hand, incongruent feedback with angry faces on WIN trials (AF-W) also activated MPFC and vACC, previously implicated in responses to social inclusion-exclusion and emotional conflict [31]–[33]. Moreover, activity in MPFC and vACC correlated with AVS and overlapped with similar regions activated by social rejection[32] or emotion suppression [24] in other paradigms, suggesting that affective evaluation processes responding to conflict situations might be more active in avoidant subjects in keeping with their more negative appraisal of others [5], [10], [25]. This might further contribute to the reduced sensitivity to social reward observed in these subjects.

In sum, our study shows that the two dimensions of adult attachment have distinct neural substrates and produce specific effects on the appraisal of social facial signals. Ventral striatum and VTA were selectively activated by the rewarding feedback value of smiling faces accompanying a success, and thus representing social reward, but this response was blunted in individuals with high AVS scores. Amygdala was selectively activated by the reproach value of angry faces combined with errors, thus representing social punishment, and this response was enhanced in individuals with high AXS scores. In other words, both striatum and amygdala responses were specific to the perceived social meaning of face expressions in relation to current task goals, because no such activity was elicited by the same expressions with a different (incongruent) feedback. Moreover, high AVS also correlated with an increased response to potential social confrontation in ACC, consistent with negative relational schemata hold by avoidant individuals. In contrast, secure attachment was characterized by higher striatal response to rewarding faces and lower amygdala responses to reproach faces, but showed no unique activation pattern, supporting the idea that it may entail a combination of low avoidant and low anxious traits [10]. By revealing a critical involvement of emotional brain systems associated with social reward and threat in adult attachment style, our fMRI data provide the first direct neurobiological evidence in support of psychological models proposing two independent affective dimensions to explain these individual differences. More generally, our data also converge with bidimensional models of social disorders that suggest distinct contributions of negative and positive emotions in regulating social behavior and interpersonal communication in a wide range of social contexts [40], [44]. Altogether, these results may ultimately help define appropriate intervention strategies in clinical disorders of attachment and social functioning, including autism, phobias, and other relational disturbances.

## Materials and Methods

### Subjects

We recruited 16 healthy volunteers (8 males, mean age 23.6±3.6, all right-handed) who had normal or corrected to normal vision, no history of neurological or psychiatric disease, and gave written informed consent according to the local ethical committee (Commission centrale d'éthique de la recherche sur l'être humain; le Comité départmental d'éthique de N.A.C.) regulation.

### Stimuli and procedure

Visual dot-counting was presented as the primary task to participants. Each trial began with a white central fixation-cross on a black screen (for 3 to 7 sec, average 3.5 sec), followed by a brief visual display divided in two parts with a variable number of white dots on each side of the screen (presented for 500 ms). The number of dots on each side ranged from 10 to 15. Their quantity and position were randomly assigned on every trial for each side separately, in such a way that the display was never visually identical on both sides (see Figure 1a). Participants had to indicate which side of the screen contained more dots (right/left) by pressing one of two response-keys. The total number of dots and the difference between the two display sides were adjusted online based on the participant's performance on preceding trials, by reducing the difference after each correct trial (minimum 1 dot) or increasing the difference after each incorrect trial (maximum 5 dots), allowing us to maintain performance close to threshold and to obtain approximately equal numbers of correct and incorrect trials (mean correct = 57±1% across conditions). In addition, to further ensure this equal distribution, occasional displays with 15 dots on both sides were inserted whenever performance exceeded 60% correct of two consecutive trials (20±5.6 out of 128 trials). None of the participants noticed these “trick” trials.

The dot display was followed by a black screen with a variable interval (jitter of 1000 to 1400 ms, average 1200 ms), during which participants gave their response; and then by a visual feedback screen (1500 ms) consisting of a face (with either a smiling or angry expression) paired with a verbal indication of actual performance on the counting task (either “WON” or “LOST”). The verbal feedback always corresponded to real performance success or failure on the preceding trial (except on the few “trick” trials with equal number of dots on both sides, where a negative “LOST” feedback was given to reduce an excess of correct over incorrect trials). By contrast, the facial emotional expression was pseudo-randomly assigned on every trial, with the constraint that smiling and angry faces appeared on an equal number of correct and incorrect trials each. This design resulted in 4 different combinations of verbal and facial feedback (see Figure 1b): Smiling Face on WON trial (SF-W) or LOST trial (SF-L), Angry Face on WON trial (AF-W) or LOST trial (AF-L). Face stimuli were colour photographs of 16 different individuals (8 males) from the Karolinska Directed Emotional Faces set (KDEF, Lundquist D., Flykt A., and Öhmann A., 1998). Each face identity was assigned to one condition only (2 males and 2 females in each of the 4 feedback types, counterbalanced across participants). Thus, for a given participant, a given face was always seen with the same expression (either smiling or angry) and the same feedback message (either positive “WON” or negative “LOST”) throughout the task. Each face identity was repeated 8 times in the corresponding conditions, in random order, resulting in 128 trials in total per participants (with a total duration of approximately 15 min).

We induced a pseudo-social game context by telling participants a cover-story along the following lines: faces were those of other subjects who already participated and belonged to two different groups; the study goal was to compare perceptual abilities and cooperation among the groups; they had been randomly assigned to play for subjects in one of these groups; each correct response gave one point to this group while each incorrect response gave one point to the other group; these outcomes would be reminded to them during the game by displaying faces from the different groups with appropriate expressions. Subsequent debriefing after scanning indicated that participants accepted the cover-story and were highly motivated by the task. They consistently reported that they were “surprised”, “irritated”, or “annoyed” by incongruent feedback combinations, and typically described these faces as “untrustworthy”, “foe”, “envious”, etc. Together with brain data, these reports clearly suggest that feedback context strongly modulated the subjective interpretation of facial expressions.

### Questionnaires

We used the Adult Attachment Questionnaire (AAQ), a validated French version [28] of the original Attachment Style Measure (ASM) [45], which includes a series of 13 statements rated along a 7-point scale (from “strongly disagree” to “strongly disagree”). This instrument yields three separate scores, one for each prototypical style including avoidant (AVS), anxious (AXS), and secure attachment (SAS), which have been shown to be reliably distinct from each other [28]. The AAQ thus provides quantitative indices for the relative strength of each of the three classic attachment categories, but also allows bi-dimensional measures for anxious and avoidant axes in attachment space. The 13 items of the ASM (or AAQ) are also included in the Relationship Scales Questionnaire (RSQ) [46], comprising 30 items in total (with some items reversed), and a recent review [29] suggests that the best method to analyze the 30-item RSQ is to rely on these 13 items alone to create a bi-dimensional attachment space made of the avoidance and anxiety measures. Moreover, we found strong correlations between avoidance and anxiety dimensions obtained by the AAQ and RSQ (Pearson r>.797, p<.0004). Other personality questionnaires included STAXI [47], STAI [48], BIS-BAS [49], and PANAS [50]. We also obtained other debriefing measures about credibility of the task and affect during the different feedback conditions using 5-point Lickert scales (e.g. degree of satisfaction or frustration on WON or LOST trials, respectively; subjective experience elicited by seeing angry or smiling faces; and subjective irritation elicited by incongruent expressions), as well as a likeability and memory test for the different face identities (these data showed no effect of attachment style and are not reported). Because the personality questionnaires from one subject were incomplete, only 15 subjects (n = 15) were included in our correlation analysis with these measures.

### MRI acquisition and analysis

MRI data were acquired on a 1.5 T whole-body INTERA system (Philips Medical Systems), using a standard head-coil configuration. For each participant, structural images were obtained with a 3D-GRE T1-weighted sequence (FOV = 250 mm, TR/TE/Flip = 15 ms/5.0 ms/30°, matrix = 256×256, slice-thickness = 1.25 mm) and functional images with a GRE EPI sequence (TR/TE/Flip = 2500 ms/40 ms/80°, FOV = 250 mm, matrix = 128×128). Functional images covered the whole brain, consisting of 30 contiguous 4mm axial slices parallel to the inferior edge of the occipital and temporal lobes, and acquired continuously for a total of 232 images per participant.

Functional images were analyzed using the general linear model for event-related designs in SPM2 (Wellcome Department of Imaging Neuroscience, London, UK; http://www.fil.ion.ucl.ac.uk/spm). All images were realigned, corrected for slice timing, normalized to an EPI template (re-sampled voxel-size of 3 mm), spatially smoothed (8 mm FWHM Gaussian kernel). A high-pass frequency filter (cutoff 120 s) and corrections for auto-correlation between scans were applied to the time series.

Statistical analysis was performed using the general linear model implemented in SPM2, with a separate regressor for each event type convolved with a canonical hemodynamic response function. Six events were modelled, including the dot display on correct and incorrect trials, and the 4 critical feedback conditions (SF-W, SF-L, AF-W, AF-L). Movement parameters from realignment corrections were entered as additional covariates of no interest to account for residual movement artifacts after realignment. Statistical parametric maps were generated from linear contrasts between the different feedback conditions in each participant. A second-stage random-effect analysis was then performed using one-sample t tests on contrast images obtained in each subject for each comparison of interest. All contrasts were performed across the whole brain using standard threshold criteria [51] of significant activation at a voxel-level of p<.001 (uncorrected) and cluster size equal or greater than 5 voxels. Average parameter estimates of activity (betas) for each feedback condition were extracted from all voxels in regions of interest (ROIs), defined by the full-extend clusters showing significant activation at a voxel-level of p<.001 (uncorrected) in the SPM group analysis (random-effect contrasts).

Statistical correlations with attachment and personality traits were performed in two stages. Firstly, we tested for a relation of the average beta values from activated ROIs with standardized questionnaire scores (attachment security, anxiety, or avoidance: SAS, AXS, and AVS, respectively) using one-tailed Pearson product moment coefficient in SPSS 14.0 (SPSS, Chicago, Illinois, United States). Secondly, whenever this first stage showed a significant correlation or a strong trend for an activated region (e.g. striatum and amygdala), we performed a whole-brain multiple regression analysis on the contrast image of interest using the relevant questionnaire scores (e.g. AVS) as a linear parametric factor in SPM2, allowing us to test for any voxels throughout the brain where activation in the given contrast varied as a function of this behavioral measure [52]. For this second-stage correlation analysis, significant effects were identified using a threshold of p<.001 at the voxel-level (uncorrected) and cluster size equal or greater than 5 voxels.

### Eye Tracker acquisition and analysis

To compare visual inspection between different conditions, eye movements were monitored continuously during scanning with an MRI-compatible infra-red eyetracker LRO L6 (Applied Science Laboratories, Bedford, MA, USA). Eye position coordinates (x and y) were recorded at 60 Hz and saved for offline analysis. Data from two subjects had to be discarded for technical reasons (inaccurate calibration or missing data). Trial-by-trial epoching and processing of the data was performed with Eyenal 6000 software (ASL, Bedford, USA). For each trial in each condition, the number and duration of fixations were calculated over two areas of interest (AOI) on the feedback screen, corresponding to the face and the word message (WON or LOST), respectively. Fixation data were averaged for each AOI, in each of the four conditions (SF-W, AF-L, AF-W, SF-L) and each participant, and then submitted to a 2×2×2 repeated-measure ANOVA using SPSS 14.0 (SPSS, Chicago, Illinois, USA). Within-subject factors were face expression (smiling or angry), success feedback (won or lost), and screen area (face or word). These analyses revealed only a significant main effect of AOI (face>word; F_1,12_ = 74.3; p<.001), indicating that subjects spent more time looking at the faces than words. Critically, there was no effect of expression, success, or any interaction. ANOVAs were also performed with a between-subject factor of “attachment style” (SAS, AXS, or AVS, as determined individually by AAQ scores), to examine whether these individual traits would modulate visual inspection times, but these analyses showed no main effect of attachment nor interaction with conditions (all Fs<1.1). These results indicate that modulation of brain responses between different attachment styles are not simply due to changes in visual attention to faces in feedback displays.
